# Spindle pole body component 25 regulates stemness of prostate cancer cells

**DOI:** 10.18632/aging.101631

**Published:** 2018-11-08

**Authors:** Feilun Cui, Huaming Tang, Jian Tan, Jianpeng Hu

**Affiliations:** 1 Department of Urology, Zhenjiang First People's Hospital, Zhenjiang 212002, China

**Keywords:** cancer stem cells (CSCs), SPC25, prostate cancer (PrC)

## Abstract

Spindle pole body component 25 (SPC25) is a component of NDC80 complex that controls spindle assembly checkpoint in the microtubule-binding domain of kinetochores. We recently showed that SPC25 is required for prostate cancer (PrC) cell proliferation and cell cycle progression, and here we investigated whether SPC25 may be a Cancer stem cell (CSC) marker in PrC. We found that the levels of SPC25 were higher in PrC samples than paired normal prostate tissue. The overall survival of PrC patients with high SPC25 was poorer than those with low SPC25. PrC cell lines were transduced with two vectors carrying a luciferase reporter and a mCherry fluorescent reporter under a cytomegalovirus promoter and a nuclear green fluorescent protein reporter under the control of a SPC25 promoter, respectively, to allow differentiating SPC25+ from SPC25- PrC cells by flow cytometry. Compared to SPC25- cells, SPC25+ cells formed significantly more tumor spheres in culture, appeared to be more resistant towards docetaxel-induced cell apoptosis, and generated larger tumors with higher frequency after serial adoptive transplantation. Thus, our data suggest that SPC25 may be highly expressed in the CSC-like cells in PrC and could be a promising target for effective treatment of PrC.

## Introduction

Prostate cancer (PrC) is the most diagnosed malignant cancer in aged Chinese men [1]. Typical PrCs are generated by small knots of malignant cells that grow in a slow speed within the prostate gland, and are well responsive to stimulation of androgen [2]. However, In rare situations, PrC cells may increase proliferation, migrate out of the prostate gland to distant tissue, and their growth can even become less dependent or independent on androgen [3], as castration-resistant prostate cancer (CRPC) [4].

Given the increasing use of prostate-specific antigen (PSA) for early diagnosis of PrC and the importance of early prognosis, intervention and treatment of PrC, it becomes extremely important to understand the molecular control of the growth of PrC [5]. Hence, identification of novel targets for regulating PrC cell proliferation and malignant alterations appears to be crucial. Cancer stem cells (CSCs) are a small portion of cancer cells that possess properties like stem cells, e.g. highly proliferative potential, tendency to metastasize and resistance to chemotherapy in the tumor mass [6]. Currently, the most effective chemotherapeutic drug for metastatic PrC is docetaxel [7]. Both surface and non-surface biomarkers have been used to identify and purify CSCs but the specificity of a certain CSC marker is limited, in which none of markers is able to purify true CSCs, but to enrich them [8]. Some markers are found to be expressed in different cancers, while some markers are only applicable in certain cancer [8]. CD133 is a well-accepted CSC-marker in PrC, although other markers like CXCR4 or features like side populations has also been used to characterize CSCs in PrC [9–11]. Since isolated “CSCs” by these markers only enriched CSC population, these purified “CSCs” actually are only CSC-like cells. Hence, identification of novel CSC markers may allow further purification of the CSC population, which is critical for CSC-based cancer therapy.

Tumorigenesis stems from genetic instability due to inaccurate chromosome segregation during the cell cycle, in which kinetochores play a central role [12]. Kinetochores contain a microtubule-binding domain to form a kinetochore-microtubule connective structure for mediating chromosome alignment to the metaphase plate, the proper progression of which is controlled by the spindle assembly checkpoint regulated by NDC80 complex [12]. The NDC80 complex comprises NDC80 (Hec1 in humans), NUF2, spindle pole body component 25 (SPC25) and SPC24, among which SPC25 was recently found upregulated in lung cancer and associated with carcinogenesis, cancer cell growth and metastasis [13]. Moreover, SPC25 was shown enriched in CSC population in lung cancer [14]. Furthermore, we recently showed that SPC25 is necessary for PrC cell proliferation and cell cycle progression [15]. Here, we investigated whether SPC25 may be a CSC marker in PrC.

We found that the levels of SPC25 were higher in PrC samples than paired normal prostate tissue. The overall survival of PrC patients with high SPC25 was poorer than those with low SPC25. PrC cell lines were transduced with two vectors carrying a luciferase reporter and a mCherry fluorescent reporter under a cytomegalovirus promoter and a nuclear green fluorescent protein reporter under the control of a SPC25 promoter, respectively, to allow differentiating SPC25+ from SPC25- PrC cells by flow cytometry. Compared to SPC25- cells, SPC25+ cells formed significantly more tumor spheres in culture, appeared to be more resistant towards docetaxel-induced cell apoptosis, and generated larger tumors with higher frequency after serial adoptive transplantation.

## RESULTS

### PrC with high SPC25 expression is associated with poor overall survival

**Figure 1:**
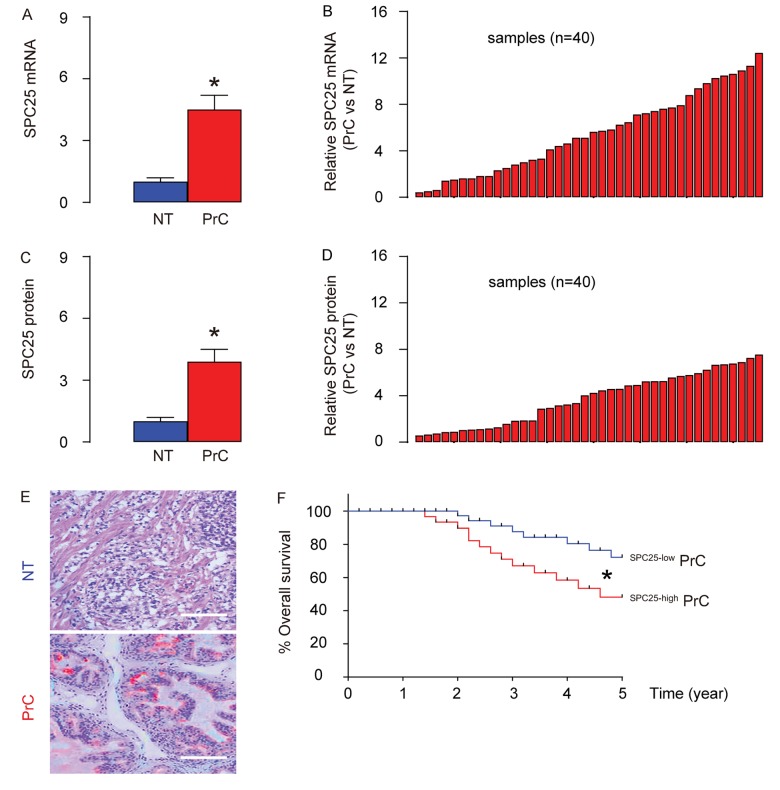
**PrC with high SPC25 expression is associated with poor overall survival.** (**A**-**B**) RT-qPCR for SPC25 mRNA levels in 40 PrC specimens and paired normal prostate tissue (NT), shown by mean ± SD (**A**), and by individual values (**B**). (**C**-**D**) ELISA for SPC25 protein levels in 40 PrC specimens and NT, shown by mean ± SD (**C**), and by individual values (**D**). (**E**) Immunostaining for SPC25 (fast red) in PrC and NT. (**F**) The 40 patients were followed-up for 5 years. The patients were classified into SPC25-high group (n=20) and SPC25-low group (n=20), based on the median value. Kaplan-Meier curves were plotted. *p<0.05. N=40. Scale bars are 100 µm.

PrC samples and paired normal prostate tissues (NT; n=40) were assessed for SPC25 mRNA and protein levels. PrC specimens expressed significantly higher mRNA levels of SPC25 than NT by RT-PCR, shown by mean± SD (Figure 1A) and by individual values (Figure 1B). Moreover, PrC specimens expressed significantly higher protein levels of SPC25 than NT by ELISA, shown by mean± SD (Figure 1C) and by individual values (Figure 1D). Increased SPC25 was also detected by immunohistochemistry in the tissue sections from PrC or NT (Figure 1E). The all 40 patients included in this study were followed up for 5 years to analyze the association between the SPC25 protein levels in the PrC specimens and the overall survival of the patients. The median value of SPC25 in the 40 patients was used as a cutoff point to separate the total samples into SPC25-high group (n=20) and SPC25-low group (n=20). The Kaplan-Meier curves for the overall 5-year survival of the PrC patients showed a significantly poorer survival for those with higher levels of SPC25 (Figure 1F). Thus, PrC with high SPC25 expression is associated with poor overall survival.

### SPC25 promotes PrC cell growth and migration

**Figure 2:**
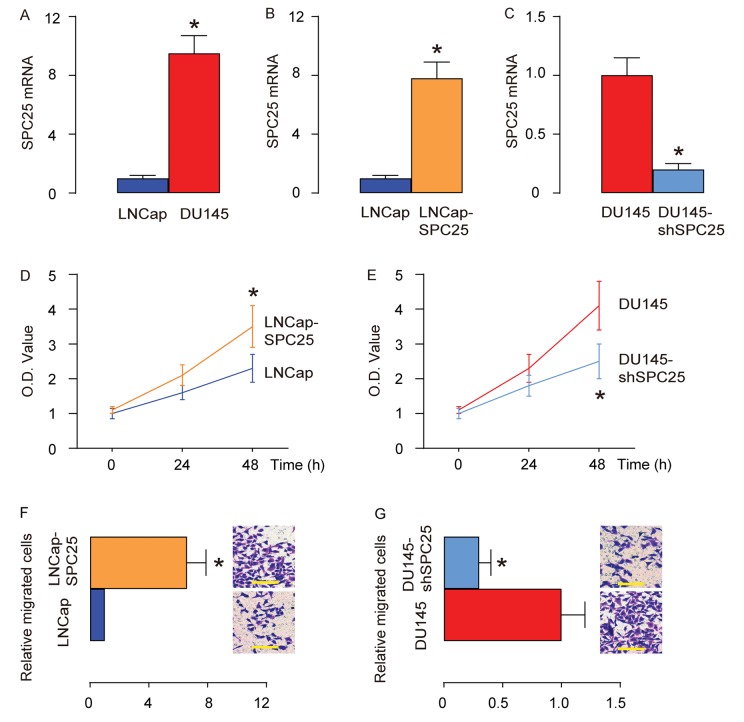
**SPC25 promotes PrC cell growth and migration.** Two human PrC cell lines (DU145 and LNCap) were used in in vitro studies. (**A**) RT-qPCR for SPC25 in DU145 and LNCap cells. (**B**) LNCap cells were transfected with a SPC25-expressing plasmid, and RT-qPCR for SPC25 was done on transfected cells, compared to control. (**C**) DU145 cells were transfected with a plasmid carrying short hairpin small interfering RNA for SPC25 (shSPC25), and RT-qPCR for SPC25 was done on transfected cells, compared to control. (**D**-**E**) MTT assay for transfected LNCap cells (**D**), and transfected DU145 cells (**E**). (**F**-**G**) Transwell cell migration assay for transfected LNCap cells (**F**), and transfected DU145 cells (**G**). *p<0.05. N=5. Scale bars are 20 µm.

Two human PrC cell lines (DU145 and LNCap) were used in in vitro studies. DU145 is androgen-insensitive due to lack of an androgen receptor, while LNCap is androgen-sensitive due to expression of an androgen receptor on the cell surface. By RT-qPCR, we found that LNCap expressed very low levels of SPC25, while DU145 expressed significantly higher levels of SPC25, compared to LNCap (Figure 2A). Thus, LNCap cells were transfected with a SPC25-expressing plasmid, and DU145 cells were transfected with a plasmid carrying short hairpin small interfering RNA for SPC25 (shSPC25), and the alteration in the SPC25 cells in the transfected LNCap (Figure 2B) and DU145 (Figure 2C) cells was confirmed by RT-qPCR. We found that overexpression of SPC25 in LNCap cells increased cell growth (Figure 2D), while depletion of SPC25 in DU145 cells decreased cell growth in an MTT assay (Figure 2E). Moreover, overexpression of SPC25 in LNCap cells increased the migrating potential of the cells (Figure 2F), while depletion of SPC25 in DU145 cells decreased the migrating potential of the cells in a transwell cell migration assay (Figure 2G). Together, these data suggest that SPC25 promotes PrC cell growth and migration.

### Differentiation of SPC25+ from SPC25- PrC cells with genetic manipulation

**Figure 3:**
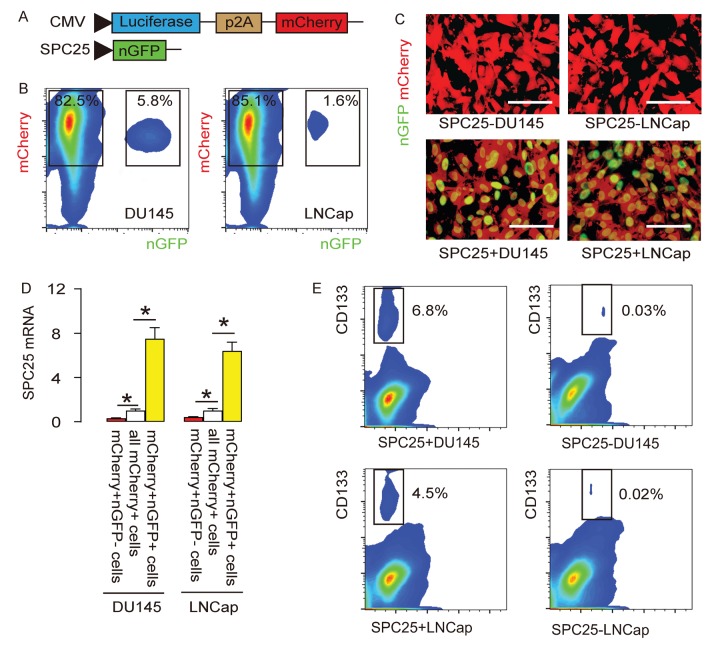
**Differentiation of SPC25+ from SPC25- PrC cells with genetic manipulation.** (**A**) The DU145 and LNCap cell lines were transduced with 2 AAVs. The first AAV carries a luciferase and a mCherry fluorescent reporter under the control of a cytomegalovirus (CMV) promotor. The luciferase and mCherry reporters are connected by a p2A sequence to allow co-expression of 2 genes by one promoter with similar efficiency. Transduction of the cells with this AAV makes the cells red fluorescent to be sortable by flow cytometry and traceable in vivo by bioluminescence assay. The second AAV carries a nuclear green fluorescent protein (nGFP) reporter under the control of a SPC25 promoter. Transduction of the cells with this AAV makes the SPC25+ cells green fluorescent in the nuclei to be sortable by flow cytometry. Co-transduction of the cells with these 2 AAVs resulted in two populations of interest. Population 1, red fluorescent (expressing mCherry but not nGFP) cells represent SPC25- cells. Population 2, yellow fluorescent (expressing both mCherry and nGFP) cells represent SPC25+ cells. (**B**) The flow cytometry analysis on infected DU145 and LNCap cells. (**C**) The purified transduced cells were examined for fluorescence in culture. (**D**) RT-qPCR for SPC25 in different cell fractions. (**E**) Flow cytometry for CD133 in SPC25+ and SPC25- fractions. *p<0.05. N=5. Scale bars were 20 µm.

In order to evaluate whether SPC25 may be a CSC marker, we designed a genetic manipulation strategy to allow separation and purification of SPC25+ vs SPC25- cells. The DU145 and LNCap cell lines were transduced with 2 AAVs. The first AAV carries a luciferase and a mCherry fluorescent reporter under the control of a cytomegalovirus (CMV) promotor. The luciferase and mCherry reporters are connected by a p2A sequence to allow co-expression of 2 genes by one promoter with similar efficiency. Transduction of the cells with this AAV makes the cells red fluorescent to be sortable by flow cytometry and traceable in vivo by bioluminescence assay. The second AAV carries a nuclear green fluorescent protein (nGFP) reporter under the control of a SPC25 promoter. Transduction of the cells with this AAV makes the SPC25+ cells green fluorescent in the nuclei to be sortable by flow cytometry. Co-transduction of the cells with these 2 AAVs resulted in two populations of interest. Population 1, red fluorescent (expressing mCherry but not nGFP) cells represent SPC25- cells. Population 2, yellow fluorescent (expressing both mCherry and nGFP) cells represent SPC25+ cells (Figure 3A). The flow cytometry analysis on infected DU145 and LNCap cells showed that most of the PrC cells were transduced and appeared to be mCherry+. Among all mCherry+ cells, there were a few cells (5.8% in DU145 or 1.6% in LNCap) appeared to be nGFP+ and thus were yellow fluorescent (Figure 3B). The red or yellow fluorescent cells were then sorted into culture and observed under fluorescent microscopy, showing consistent results with flow cytometry-based cell sorting (FACS, Figure 3C). Next, we assessed the SPC25 mRNA levels in nGFP+mCherry+ and nGFP- mCherry+ cells, compared to total mCherry+ cells, suggesting that the labeling technology has faithfully fulfilled the correct separation of SPC25+ cells vs SPC25- cells (Figure 3D). Since CD133 is a well-accepted CSC marker in PrC, we analyzed CD133 positivity in SPC25+ and SPC25- populations. Significantly higher percentage of CD133+ cells were detected in SPC25+ fraction than in SPC25- fraction in both lines (Figure 3E). Together, these data suggest that this dual transduction system successfully separates SPC25- cells from SPC25+cells and the latter may include significantly higher number of CSCs.

### SPC25+ PrC cells are enriched with CSCs

**Figure 4:**
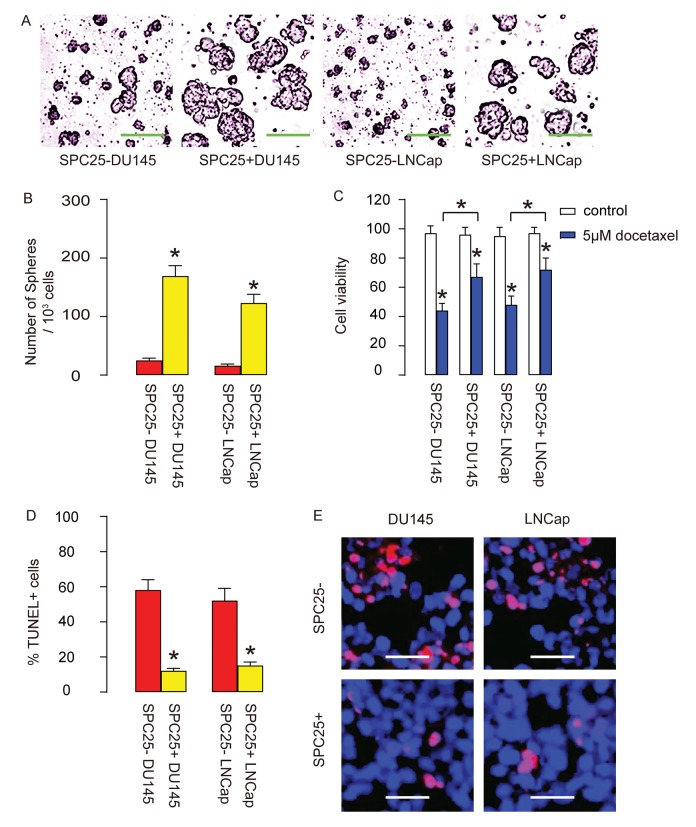
**SPC25+ PrC cells are enriched with CSCs.** (**A**-**B**) The tumor sphere formation by SPC25- and SPC25+ cells from two cell lines, shown by representative images (**A**) and by quantification (**B**). (C-E) Docetaxel was applied to SPC25- and SPC25+ cells from both cell lines. (**C**) Cell viability by CCK-8 assay. (**D**-**E**) Cell apoptosis by TUNEL assay, shown by quantification (**D**), and by representative images (**E**). *p<0.05. N=5. Scale bars in A were 50 µm and in **E** were 20 µm.

The in vitro features for CSCs include tumor sphere formation and resistance to chemotherapeutic drugs. Thus, SPC25+ cells and SPC- cells from DU145 and LNCap cells were assessed by these two methods. Firstly, tumor sphere formation was tested, showing that significantly more tumor spheres were formed in SPC25+ cells, compared to SPC25- cells, shown by representative images (Figure 4A), and by quantification (Figure 4B). Next, docetaxel was applied to SPC25- and SPC25+ cells from DU145 and LNCap cells respectively, and the cell survival was assessed with CCK-8 assay, showing significantly better cell survival in SPC25+ cells compared to SPC25- cells (Figure 4C). The compromised cell survival appeared to result from cell apoptosis by TUNEL assay, showing significantly less TUNEL+ cells in SPC25+ fraction compared to SPC25- fraction, shown by quantification (Figure 4D), and by representative images (Figure 4E). Together, these data suggest that SPC25+ PrC cells presented with CSC features in vitro.

### SPC25+ PrC cells present CSC features in vivo

**Figure 5:**
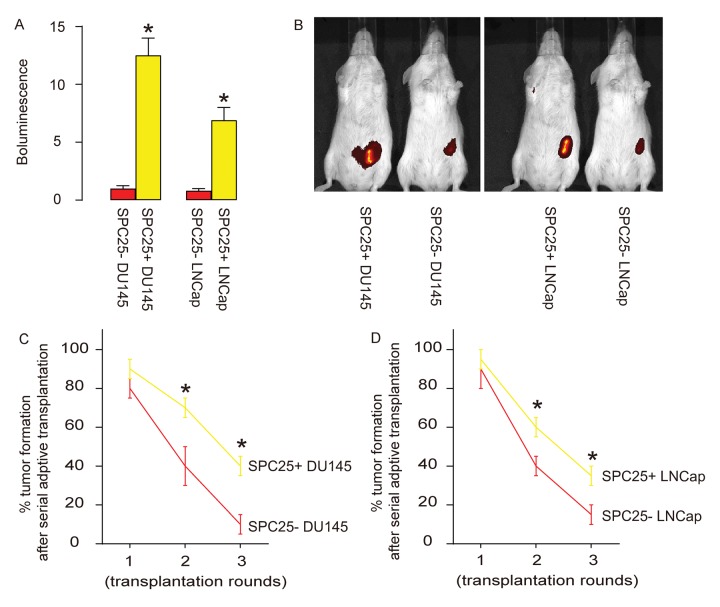
**SPC25+ PrC cells present CSC features in vivo.** (**A**-**B**) Same number of SPC25- or SPC25+ PrC cells from both DU145 and LNCap lines were subcutaneously transplanted into nude mice and assess for the tumor formation by bioluminescence after 8 weeks, shown by representative images (**A**), and by quantification (**B**). (**C**-**D**) Twenty cells were purified from the tumor developed from implanted SPC25- or SPC25+ cells from DU145 (**C**) and LNCap (**D**) lines were subcutaneously transplanted back to new nude mice. A total of 3 rounds of serial adoptive transplantations were performed. The frequency of tumor formation was shown. p<0.05. N=10.

Same number of SPC25- or SPC25+ PrC cells from both DU145 and LNCap lines were subcutaneously transplanted into nude mice and assess for the tumor formation by bioluminescence after 8 weeks. Significantly larger tumors were observed in SPC25+ cells-transplanted mice, compared to SPC25- cells-transplanted mice, shown by representative images (Figure 5A), and by quantification (Figure 5B). Serial adoptive transplantation of SPC25+ and SPC25- cells to form tumor was also assessed. Twenty cells were purified from the tumor developed from implanted SPC25- or SPC25+ cells were subcutaneously transplanted back to new nude mice. A total of 3 rounds of serial adoptive transplantations were performed, showing more frequent tumor formation in SPC25+ cell-transplanted mice, compared to SPC25- cell-transplanted mice (Figure 5C-D). Together, these data suggest that SPC25+ PrC cells present CSC features in vivo.

## DISCUSSION

Although some specific markers have been used to identify or purify CSCs, e.g. CD133 or CXCR4 in PrC [9–11], it is gradually realized that a single marker or combined a few markers is not sufficient to allow definition and purification of CSCs in tumor [16]. Recently, characterization of CSCs in PrC has been improved with the increasing use of next-generation sequencing (NGS) technology, from which we got to know that CSCs are characterized with expression of clusters of genes that regulate cell cycle, stress, apoptosis, autophagy, nutrient usage, etc [17]. However, data from NGS need to be confirmed by traditional methods for characterizing CSCs.

The association between SPC25 and tumorigenesis was just reported very recently. From the limited supply of literature, upregulation of SPC25 was consistently detected in lung cancer [13,14] and in PrC [15]. In addition, Chen et al. has shown that SPC25 regulates stemness of lung cancer cells [14]. However, the gold standards to identify CSCs were not used in Chen’s study, probably due to the fact that SPC25 is not a surface marker that could be easily used to differentiate positive vs negative populations [14]. Therefore, we addressed this question in PrC with a genetic modification strategy. In this approach, all cancer cells were labeled with a red fluorescent protein, mCherry, but only those SPC25+ cells were co-labeled with a nGFP to be expressing also green fluorescent. Co-expression of both mCherry and nGFP made the SPC25+ cancer cells appear to be yellow fluorescent, and thus allow separation of them from SPC25- cancer cells by flow cytometry. Gene profiling and direct fluorescence examining validated this strategy. Similar approaches have been used in some previous studies with success [18–20].

Here, SPC25 was found to contribute to stemness of PrC in both androgen-sensitive cell line and androgen-insensitive cell line, which suggests that SPC25 as a therapeutic target may be used for both castration-sensitive prostate cancer (CSPC) and CRPC [4]. It is very important since CRPC currently has no effective treatments and will benefit from discovering new targets [4].

The clinical data for SPC25 in previous studies were primarily obtained from the cancer genome atlas (TCGA, NIH, USA) [13–15]. Nevertheless, the current analysis was based on our own data from the patients administrated in our hospitals. Hence, these clinical evidence for upregulation of SPC25 provide more information and confidence of the conclusion, given the relative diffuse sources for data in TCGA. A previous study also showed that SPC25 levels are critical for proper spindle checkpoint activation of a cell cycle, which means that a modest upregulation of SPC25 may promote the progression of a cell cycle, while extremely high levels of SPC25 may lead to cell cycle arrest [17]. In the current study, the experimental alteration of SPC25 levels in PrC cells was modest and comparable with the changes in clinical specimens. Therefore, it may be interesting to assess the effects of extremely high levels of SPC25 on PrC cell growth, metastasis, chemo-resistance and stemness in future studies.

In conclusion, our study provided evidence that SPC25+ could be a specific CSC marker in PrC. The outcome of current PrC therapy, especially CRPC, could be improved through treatments that selectively target SPC25.

## MATERIALS AND METHODS

### Objective

The PrC and paired normal prostate tissue from 40 patients were collected in Zhenjiang First People's Hospital from 2007 through 2012. The survivals of these patients were followed up for 5 years. Written approval was obtained from all the involved patients who have been informed of the research purpose and procedure. Experiments and animal work have been approved by the institutional review board of the Zhenjiang First People's Hospital.

### Cell culture and treatment

Two human PrC cell lines (LNCap and DU145) were obtained from American Type Culture Collection (ATCC, Rockville, MD, USA). These cells were maintained in DMEM (Invitrogen, Rockville, MD, USA) suppled with 10% fetal bovine serum (FBS; Sigma-Aldrich, Rockville, MD, USA) in a humidified incubator at 37 °C with a 5% CO_2_. Docetaxel (Sigma-Aldrich) was prepared in a stock of 1 mM and applied to cells in the concentration of 5 µM. Analysis was done 24 hours after treatment.

### Cell transfection and transduction

Complete coding sequence for SPC25 was obtained by PCR using human PrC cell line DU145 as a template. The sequence for small hairpin RNA (shRNA) targeting human SPC25 was 3'‑CCGGCCATCAAAGCATTTGCAGAAACTCGAGTTTCTGCAAATGCTTTGATGGTTTTTG‑5', as described [15]. Scrambled shRNAs were used as negative controls. After annealing, all oligonucleotides were inserted to pCDNA3 plasmid (GenePharma, Shanghai, China). Transfection of the cells was performed using Lipofectamine 3000 (Invitrogen). The PrC cell lines were co-transduced with 2 adeno-associated viruses (AAVs). The first AAV carries a luciferase and a mCherry fluorescent reporter under the control of a cytomegalovirus (CMV) promotor. The luciferase and mCherry reporters are connected by a p2A sequence to allow co-expression of 2 genes by one promoter with similar efficiency. The second AAV carries a nuclear green fluorescent protein (nGFP) reporter under the control of a SPC25 promoter. For generating AAVs, a pAAV-CMV-nGFP plasmid and a pAAV-CMV-luciferase-p2A-mCherry plasmid were used as backbones (Clontech, Rockville, MD, USA). SPC25 promoter was generated by PCR using genomic DNA of human fibroblast as the template. Prepared plasmids, serotype 8 helper plasmids and packaging plasmids were used to co-transfect HEK293T cells (ATCC) using Lipofectamine-3000 assay, followed by purification and titration. In vitro transduction of PrC cell lines used a multiplicity of infection (MOI) of 120 and a 72 hours’ incubation.

### Flow cytometry

Flow cytometry was applied to purify cells. GFP and mCherry were analyzed by direct fluorescence. For CD133, a PE-cy7 anti-human CD133 antibody (Becton-Dickinson Biosciences, San Jose, CA, USA) was used.

### Animal experiments

The male nude mice at 10-week-old (SLAC Laboratory Animal Co. Ltd, Shanghai, China) were used for subcutaneous implantation of PrC cells. For serial adoptive transfer, 20 cells from the formed tumor in the previous round were used. The tumor formation was monitored and quantified with bioluminescence (IVIS imaging system, USA).

### Tumor sphere formation

The purified PrC cells were cultured in tumor sphere media (R&D Systems, Shanghai, China) at a density of 1.5X10^4^ cells per well. The tumor sphere media are consisting of a serum-free DMEM, 20 ng/ml human recombinant epidermal growth factor, 20 ng/ml basic fibroblast growth factor, 10 ng/ml leukemia inhibitory factor and 60 µg/ml N-acetylcysteine. The tumor sphere was observed and assessment one week after seeding.

### Cell proliferation and viability assay

The cell proliferation was assessed by using an MTT Kit (Roche, Indianapolis, IN, USA), in which the quantification was done at 540nm absorbance value (OD) from 5 repeats. The cell viability was detected with Cell Counting Kit-8 (CCK-8) assay and quantified at OD=450nm, according to the manufacture’s instruction (Sigma-Aldrich). All experiments were performed in duplicate.

### TUNEL assay

The terminal deoxynucleotidyl  transferase (TdT)-mediated dUTP nick-end labeling (TUNEL) was performed using a cy5-TUNEL staining kit (R&D Systems). The positive controls used 10 minutes’ incubation at room temperature with 1500 U/ml DNAse1 in 50 mM Tris pH 7.5, 10 mM MgCl_2_ and 1 mg/ml BSA.

### Real time quantitative PCR (RT-qPCR)

The RT-qPCR was applied using the commercial SYBR Green PCR Kit (Qiagen, Shanghai, China) and the designed primers. The primers for quantifying SPC25 are 5'‑AGAAGAACGAATGGTTGAGAT‑3' (forward) and 5'‑TCCTGGATATTTGCAGTCAGT‑3' (reverse); The primers for quantifying β-actin: 5’-CTCTTCCAGCCTTCCTTCCT-3’ (forward) and 5’-AGCACTGTGTTGGCGTACAG-3’ (reverse). The RT-qPCR reactions were performed in duplicate. The levels of gene expression were quantified with the 2-△△Ct method and normalized sequentially with β-actin and the experimental controls.

### ELISA

Total protein was extracted from the cells using RIPA buffer (Sigma-Aldrich) and quantified with BCA protein assay (Sigma-Aldrich). ELISA was performed using an anti-human SPC25 ELISA kit (MyBiosource, MBS9328421, San Diego, CA, USA) as instructed.

### Cell migration assay

Cells were suspended in serum-free medium and then seeded in the top chamber of a transwell insert (Millipore, Bedford, MA, USA). The lower chamber was filled with medium supplemented with 10% FBS. After 48 hours, the cells that had migrated to the lower surface were fixed with methanol, stained with crystal violet, and counted.

### Statistical analysis

The data was statistically analyzed with GraphPad Prism 7 (GraphPad, Chicago, IL, USA). The Student’s T test was performed to compare the data of 2 groups. The values were expressed as mean ± standard deviation (SD). When p<0.05, the data was considered as significant. Kaplan-Meier curve was applied to record the overall survival of the patients included in this study.

## References

[1] Fonseka LN, Kallen ME, Serrato-Guillen A, Chow R, Tirado CA. Cytogenetics and Molecular Genetics of Prostate Cancer: A Comprehensive Update. J Assoc Genet Technol. 2015; 41:100–11. 26606177

[2] Stuchbery R, McCoy PJ, Hovens CM, Corcoran NM. Androgen synthesis in prostate cancer: do all roads lead to Rome? Nat Rev Urol. 2017; 14:49–58. 10.1038/nrurol.2016.221 27824348

[3] Krušlin B, Ulamec M, Tomas D. Prostate cancer stroma: an important factor in cancer growth and progression. Bosn J Basic Med Sci. 2015; 15:1–8. 10.17305/bjbms.2015.449 26042506 PMC4469930

[4] Shen MM, Abate-Shen C. Molecular genetics of prostate cancer: new prospects for old challenges. Genes Dev. 2010; 24:1967–2000. 10.1101/gad.1965810 20844012 PMC2939361

[5] Donkena KV, Yuan H, Young CY. Recent advances in understanding hormonal therapy resistant prostate cancer. Curr Cancer Drug Targets. 2010; 10:402–10. 10.2174/156800910791208544 20464780

[6] Zhou J, Ma X, Bi F, Liu M. Clinical significance of circulating tumor cells in gastric cancer patients. Oncotarget. 2017; 8:25713–20. 10.18632/oncotarget.14879 28147337 PMC5421964

[7] Vale CL, Burdett S, Rydzewska LH, Albiges L, Clarke NW, Fisher D, Fizazi K, Gravis G, James ND, Mason MD, Parmar MK, Sweeney CJ, Sydes MR, et al, and STOpCaP Steering Group. Addition of docetaxel or bisphosphonates to standard of care in men with localised or metastatic, hormone-sensitive prostate cancer: a systematic review and meta-analyses of aggregate data. Lancet Oncol. 2016; 17:243–56. 10.1016/S1470-2045(15)00489-1 26718929 PMC4737894

[8] Wang T, Shigdar S, Gantier MP, Hou Y, Wang L, Li Y, Shamaileh HA, Yin W, Zhou SF, Zhao X, Duan W. Cancer stem cell targeted therapy: progress amid controversies. Oncotarget. 2015; 6:44191–206. 10.18632/oncotarget.6176 26496035 PMC4792551

[9] Zhou J, Wang H, Cannon V, Wolcott KM, Song H, Yates C. Side population rather than CD133(+) cells distinguishes enriched tumorigenicity in hTERT-immortalized primary prostate cancer cells. Mol Cancer. 2011; 10:112. 10.1186/1476-4598-10-112 21917149 PMC3180433

[10] Vander Griend DJ, Karthaus WL, Dalrymple S, Meeker A, DeMarzo AM, Isaacs JT. The role of CD133 in normal human prostate stem cells and malignant cancer-initiating cells. Cancer Res. 2008; 68:9703–11. 10.1158/0008-5472.CAN-08-3084 19047148 PMC3072758

[11] Miki J, Furusato B, Li H, Gu Y, Takahashi H, Egawa S, Sesterhenn IA, McLeod DG, Srivastava S, Rhim JS. Identification of putative stem cell markers, CD133 and CXCR4, in hTERT-immortalized primary nonmalignant and malignant tumor-derived human prostate epithelial cell lines and in prostate cancer specimens. Cancer Res. 2007; 67:3153–61. 10.1158/0008-5472.CAN-06-4429 17409422

[12] Tooley J, Stukenberg PT. The Ndc80 complex: integrating the kinetochore’s many movements. Chromosome Res. 2011; 19:377–91. 10.1007/s10577-010-9180-5 21311965 PMC3148027

[13] Jeong J, Keum S, Kim D, You E, Ko P, Lee J, Kim J, Kim JW, Rhee S. Spindle pole body component 25 homolog expressed by ECM stiffening is required for lung cancer cell proliferation. Biochem Biophys Res Commun. 2018; 500:937–43. 10.1016/j.bbrc.2018.04.205 29709477

[14] Chen J, Chen H, Yang H, Dai H. SPC25 upregulation increases cancer stem cell properties in non-small cell lung adenocarcinoma cells and independently predicts poor survival. Biomed Pharmacother. 2018; 100:233–39. 10.1016/j.biopha.2018.02.015 29432994

[15] Cui F, Hu J, Fan Y, Tan J, Tang H. Knockdown of spindle pole body component 25 homolog inhibits cell proliferation and cycle progression in prostate cancer. Oncol Lett. 2018; 15:5712–20. 29552205 10.3892/ol.2018.8003 PMC5840511

[16] Brown DV, Mantamadiotis T. Insights into the next generation of cancer stem cell research. Front Biosci. 2014; 19:1015–27. 10.2741/4264 24896333

[17] Li CW, Chen BS. Investigating core genetic-and-epigenetic cell cycle networks for stemness and carcinogenic mechanisms, and cancer drug design using big database mining and genome-wide next-generation sequencing data. Cell Cycle. 2016; 15:2593–607. 10.1080/15384101.2016.1198862 27295129 PMC5053590

[18] Cui H, Yang S, Jiang Y, Li C, Zhao Y, Shi Y, Hao Y, Qian F, Tang B, Yu P. The glycosyltransferase ST6Gal-I is enriched in cancer stem-like cells in colorectal carcinoma and contributes to their chemo-resistance. Clin Transl Oncol. 2018; 20:1175–84. 10.1007/s12094-018-1840-5 29423671

[19] Song W, Li Q, Wang L, Huang W, Wang L. FoxO1-negative cells are cancer stem-like cells in pancreatic ductal adenocarcinoma. Sci Rep. 2015; 5:10081. 10.1038/srep10081 26068418 PMC4464177

[20] Zou S, Wang C, Liu J, Wang Q, Zhang D, Zhu S, Xu S, Kang M, He S. Sox12 Is a Cancer Stem-Like Cell Marker in Hepatocellular Carcinoma. Mol Cells. 2017; 40:847–54. 10.14348/molcells.2017.0129 29127951 PMC5712514

